# The chicken and egg problem: CGRP release due to trigeminal activation or vice versa?

**DOI:** 10.1177/03331024211042360

**Published:** 2021-10-04

**Authors:** Karl Messlinger

**Affiliations:** Institute of Physiology and Pathophysiology, Friedrich-Alexander-University, Erlangen-Nürnberg Universitätsstr. Erlangen, Germany

Calcitonin gene-related peptide (CGRP) has long been in the focus of migraine research as an indicator but also as a key mediator involved in the generation and aggravation of migraine attacks (1) but its precise mechanisms in peripheral nociceptive signalling and central neurotransmission are based on speculation rather than clear knowledge. CGRP is phylogenetically an old neuropeptide (2) and is present in nearly all organs, where its main function is to support blood perfusion in critical situations (3). However, in the trigeminovascular system, which is regarded as the anatomical basis for headache generation (4,5), it seems to play a special role (6). CGRP is not immediately painful in any tissue but seems to booster sensitization of trigeminal afferents (7), and its infusion causes migraine-like pain in most migraineurs, delayed by hours (8,9). The finding that CGRP concentrations are increased in the venous outflow from the head during migraine and normalized after successful migraine treatment (8) has spurred the efforts to use CGRP as a biomarker for migraine states and types. Apart from jugular or antecubital venous plasma or serum (10–12), saliva (13,14) and recently tear fluid (15) have been examined for their CGRP content by radioimmunoassay or ELISA and found useful to define migraine states, albeit with different success (16,17).

In the present longitudinal pioneering study, the group of Patricia Pozo-Rosich used saliva to measure CGRP levels in 22 females suffering from episodic migraine of different frequency compared to 22 healthy controls (18). They applied the strategy of close-meshed sampling every day during one month and included additional samples during migraine attacks. In this way, differences in CGRP levels both between patients and controls as well as during the migraine cycle of the individuals could be assessed. The highly individual CGRP levels known from previous studies required sophisticated statistical techniques to yield usable results. In short, the median interictal salivary level was 98 pg/mL in migraineurs compared to 54 pg/mL in controls, which was significantly different, while plasma levels (6 pg/mL vs. 5 pg/mL) were much lower and not different. One day prior to the migraine attack salivary CGRP levels were 169 pg/mL rising to 247 pg/mL during the beginning of headache and returned towards previous levels already two hours after headache onset. Both, interictal CGRP levels and the magnitude of increase, were clearly higher in patients with higher migraine frequency. It appears somewhat strange that CGRP levels directly before and after migraine attacks were above those found interictally and raise the question if there was already an increase towards the attack, but changes in the course of the whole migraine cycle have not been reported by the authors. Another result, which may be critically seen by the readers since it appears as a circular argument, is the classification of patients into those with a high (significant) increase in saliva CGRP levels (called “CGRP dependent”, about 80%) and those with no increase (“non-CGRP dependent, about 20%), inasmuch as in some patients with more than one attack during the observation time, both these responses occurred. The significance of this observation should not be overestimated and is only justified in the light of a similar ratio of migraineurs who respond to CGRP infusion with delayed migraine-like attacks (9), and also because there was significant association of photophobia and phonophobia with the “CGRP dependent” group. Thus a more extended study including more participants over a prolonged observation time, possibly combined with a CGRP provocation test, appears essential to clarify this point.

In comparison to CGRP measurements in other compartments like plasma or tear fluid, sampling and assessment of the peptide in salivary appears elegant and easy to repeat, however, the method requires high compliance of participants, since it can be confounded by several factors and depends on careful handling and extraction protocols. The observation that the CGRP concentration in saliva is higher compared to circulating plasma CGRP may indicate a concentrated release from trigeminal afferents which sparsely innervate the salivary glands (19,20) and also excludes the idea that CGRP is taken up secondarily from the facial circulation into the saliva. The high salivary levels instead indicate that CGRP is not only released from intracranial perivascular afferents. This is perplexing insofar as these structures are thought to be particularly responsible for the migraine headache (21,22), while facial pain phenomena are restricted at most to hypersensitivity (5), and intraoral pain is not a feature of migraine. Thus an increased activity in migraine may be assumed for the whole trigeminal system indicated by high CGRP release – again CGRP merely as a bioindicator? 

In conclusion, the present study is relevant by mainly reviving some decisive questions about the effects of CGRP in the trigeminovascular system and in migraine: What comes first: trigeminal afferent activation or CGRP release? Is CGRP released from trigeminal afferents and drives the pain phase of migraine attacks, or is it just a bioindicator for vigorous activation of trigeminal afferents? Can migraine attacks occur without head pain though with increased CGRP release? Is the CGRP release from peripheral or central trigeminal structures also responsible for neurological symptoms like photo- and phonophobia and how does this occur? Are there indeed different types of migraine, dependent on CGRP levels and possibly other peptides of the calcitonin family or non-peptide mediators like nitric oxide? And finally, can salivary CGRP levels be used to stratify patients and their anti-migraine treatment targeting the CGRP signalling system?
